# Physiological MRI Biomarkers in the Differentiation Between Glioblastomas and Solitary Brain Metastases

**DOI:** 10.1007/s11307-021-01604-1

**Published:** 2021-04-23

**Authors:** Elisabeth Heynold, Max Zimmermann, Nirjhar Hore, Michael Buchfelder, Arnd Doerfler, Andreas Stadlbauer, Natalia Kremenevski

**Affiliations:** 1 Department of Neurosurgery, Friedrich-Alexander University (FAU) Erlangen-Nürnberg, Schwabachanlage 6, 91054 Erlangen, Germany; 2 Department of Preclinical Imaging and Radiopharmacy, University of Tübingen, Röntgenweg 13, 72076 Tübingen, Germany; 3 Department of Neuroradiology, Friedrich-Alexander University (FAU) Erlangen-Nürnberg, Schwabachanlage 6, 91054 Erlangen, Germany; 4 Institute of Medical Radiology, University Clinic of St. Pölten, Karl Landsteiner University of Health Sciences, Dunant Platz 1, St. Pölten, Austria

**Keywords:** Glioblastoma, Brain metastasis, Physiological MRI, Hypoxia, Angiogenesis

## Abstract

**Purpose:**

Glioblastomas (GB) and solitary brain metastases (BM) are the most common brain tumors in adults. GB and BM may appear similar in conventional magnetic resonance imaging (cMRI). Their management strategies, however, are quite different with significant consequences on clinical outcome. The aim of this study was to evaluate the usefulness of a previously presented physiological MRI approach scoping to obtain quantitative information about microvascular architecture and perfusion, neovascularization activity, and oxygen metabolism to differentiate GB from BM.

**Procedures:**

Thirty-three consecutive patients with newly diagnosed, untreated, and histopathologically confirmed GB or BM were preoperatively examined with our physiological MRI approach as part of the cMRI protocol.

**Results:**

Physiological MRI biomarker maps revealed several significant differences in the pathophysiology of GB and BM: Central necrosis was more hypoxic in GB than in BM (30 %; *P* = 0.036), which was associated with higher neovascularization activity (65 %; *P* = 0.043) and metabolic rate of oxygen (48 %; *P* = 0.004) in the adjacent contrast-enhancing viable tumor parts of GB. In peritumoral edema, GB infiltration caused neovascularization activity (93 %; *P* = 0.018) and higher microvascular perfusion (30 %; *P* = 0.022) associated with higher tissue oxygen tension (33 %; *P* = 0.020) and lower oxygen extraction from vasculature (32 %; *P* = 0.040).

**Conclusion:**

Our physiological MRI approach, which requires only 7 min of extra data acquisition time, might be helpful to noninvasively distinguish GB and BM based on pathophysiological differences. However, further studies including more patients are required.

**Supplementary Information:**

The online version contains supplementary material available at 10.1007/s11307-021-01604-1.

## Introduction

Glioblastomas (GB) and solitary brain metastases (BM) constitute two of the most frequently diagnosed types of brain tumors in adults [1]. The annual incidence of GB in the population is 4–5/100,000 people. BM on the other hand occurs in 10–30 % of patients with metastatic cancer as the first manifestation of disease [2]. Giordana et al. [3] showed in their study that approximately 55 % patients with newly diagnosed solitary brain lesions had no known history of malignancy at the time of diagnosis. GB is characterized as a highly aggressive and rapidly progressive brain tumor. The median survival time is only 60 weeks, despite advanced multimodal treatment with radical surgery followed by combined adjuvant radiotherapy and chemotherapy [4]. BM is also associated with a dismal prognosis and approximately less than 2 months median survival of patients with untreated BM [5]. Nevertheless, maximum possible surgical cytoreduction followed by adjuvant radiotherapy and chemotherapy can significantly extend the overall survival of these patients. The clinical approach and management strategy of GB and BM are quite different for each tumor type and can have a significant consequence on the clinical outcome. The current gold standard of GB management comprises of primary surgery with an aim to attain gross total resection as defined by a minimum of 98% reduction in visible tumor volume followed by combined adjuvant treatment consisting of fractioned stereotactic radiation and concomitant oral chemotherapy with temozolomide [6]. On the other hand, in case of suspected BM, initially a CT staging to possibly locate the primary tumor and determine the extent of metastatic disease is necessary. Surgical management of BM then depends on number and location. Adjuvant treatment then comprises of either stereotactic fractionated or whole brain radiation accompanied by systemic chemotherapy [7].

Due to its excellent soft tissue visualization, magnetic resonance imaging (MRI) has already been established as the noninvasive diagnostic modality of choice and as an essential part of routine clinical work up in the diagnosis and evaluation of brain tumors. However, the fact that both GB and BM may have similar imaging characteristics in conventional MRI scans—a space occupying lesion with ring-like contrast enhancement, central necrosis, and surrounding perifocal edema—represents a great challenge in clinical practice, mandating primary surgical management to ascertain histological dignity [8]. Recently developments in MRI methodology enable the assessment of physiological and metabolic properties of various tissues including the tumor microenvironment [9].

It is well known that tumor progression and growth depend on an extensive of oxygen and nutrient supply for energy production. New vascular networks are formed to meet the increased metabolic demand as well as to provide roads for tumor cell migration. GB is an aggressive tumor with a unique microvascular proliferation leading to extensive vascularization with high microvascular density and heterogeneous perfusion [10]. These markedly abnormal blood vessels are structurally irregular, more tortuous, disorganized, and fragile with either increased blood-brain barrier permeability or varying degrees of disruption. The immature vascular networks together with the aggressive tumor growth exceeding the blood supply lead to the creation of the necrotic and hypoxic zone in GB [11]. On the other hand, capillaries in BM are characterized by an absence of blood-brain barrier cells and prominent capillary fenestration. The microvascular structure mirrors that of the site of the primarius cancer. In summary, GB is characterized by infiltrative growth with invasion of surrounding brain tissue, whereas BM is characterized by expansive growth with displacement of adjacent brain tissue.

We and others have recently demonstrated that novel MRI techniques including quantitative blood oxygenation level dependent (qBOLD) imaging and vascular architecture mapping (VAM) provide valuable pathophysiological and metabolic information on malignant tumors [9]. These new imaging biomarkers enable noninvasive quantitative measurement of tissue hypoxia, microvascular vessel diameter, and neovascularization activity, thereby providing precise insight into the heterogeneously structured tumor microenvironment [10, 12].

Thus, the aim of this study was to determine whether the imaging biomarkers of hypoxia, microvascular architecture, and neovascularization activity can be of assistance in differentiating GB from BM in patients with solitary enhancing brain mass with similar MRI signal alterations.

## Materials and Methods

### Patients

A consecutively and prospectively populated institutional database was searched for patients with newly diagnosed, untreated brain tumors between August 2017 and June 2020. Inclusion criteria were as follows: (i) age ≥18 years; (ii) our study MRI protocol was included in the MRI scan for initial diagnosis; and (iii) histopathological confirmation of either a glioblastoma (GB) based on the World Health Organization (WHO) histological grading system or a brain metastasis (BM). The institutional review board approved this retrospective analysis. All patients gave their written informed consent permitting scientific work with their clinical data and MRI scans.

### MRI Data Acquisition

MRI data acquisition was carried out on a 3-T whole-body scanner (Trio, Siemens, Erlangen, Germany) equipped with the standard 12-channel head coil. The conventional MRI (cMRI) protocol for diagnosis of brain tumors in clinical routine included, among others, the following sequences: (i) an axial fluid-attenuated inversion-recovery (FLAIR; TR/TE/TI: 5000/460/1800 ms; in-plane resolution: 0.45 × 0.45 mm, slice thickness: 3 mm; 48 slices); (ii) an axial diffusion-weighted imaging (DWI) sequence (TR/TE: 5300/98 ms; in-plane resolution: 1.2 × 1.2 mm, slice thickness: 4 mm; 29 slices, *b*-values: 0 and 1000 s/mm2); (iii) pre- and post-contrast enhanced (CE) high-resolution three-dimensional (3D) T1-weighted magnetization-prepared rapid acquisition with gradient echo (MPRAGE) sequences (TR/TE: 2100/2.3 ms; in-plane resolution: 1.0 × 1.0 mm, slice thickness: 1 mm; 176 slices); and (iv) an axial gradient echo (GE) dynamic susceptibility contrast (DSC) perfusion MRI sequence (TR/TE: 1740/22 ms; in-plane resolution: 1.8 × 1.8 mm, slice thickness: 4 mm; 29 slices) with 60 dynamic measurements during administration of 0.1 mmol/kg bodyweight gadoterate-meglumine (Dotarem, Guerbet) at a rate of 4 ml/s using a MR-compatible injector (Spectris, Medrad). A 20-ml bolus of saline was injected subsequently at the same rate.

For MRI-based examination of microvascular architecture and neovascularization activity using the vascular architecture mapping (VAM) approach, we performed a DSC perfusion MRI sequence obtained with a SE echo-planar imaging read out (SE-DSC) using the same geometric parameters, slice position, and contrast agent injection protocol as applied for the routine GE-DSC perfusion MRI. Our strategies to minimize patient motion and differences in time to first-pass peak, which may significantly affect the data evaluation, were described previously [9].

For MRI-based investigation of tissue oxygen metabolism using the quantitative blood oxygen level depended (qBOLD) approach, we performed a multi-echo SE sequence (TR/TE: 3260/13–104 m; 8 echoes) and a multi-echo GE sequence (TR/TE: 1210/5–40 ms; 8 echoes) for quantitative mapping of the transverse relaxation rates R2 (= 1/T2) and R2* (= 1/T2*), respectively. All experimental sequences for VAM and qBOLD used the same geometric parameters (voxel size, number of slices, etc.) and identical slice position as was used for the routine GE-DSC perfusion sequence. The additional data acquisition time (TA) for VAM (SE-DSC perfusion: TA, 2 min) and qBOLD (R2- and R2*-mapping: TA, 3.5 and 1.5 min, respectively) was in total 7 min.

### MRI Data Processing

Processing of VAM and qBOLD data as well as calculation of MRI biomarker maps was performed with custom-made MatLab (MathWorks, Natick, MA) software. Details of the MRI data processing pipeline were published previously [9, 13–15] and are described in Suppl. Figure 1, respectively (see ESM). Briefly, VAM processing consisted of five steps starting with (i) correction for remaining contrast agent extravasation and (ii) fitting of the first bolus curves for each voxel of the GE- and SE-DSC perfusion data with a gamma-variate function. The fits were used for (iii) determination of the ∆R2, GE versus (∆R2,SE)3/2 diagram, the so-called vascular hysteresis loop (VHL) [9, 10]. VHLs, in turn, were used for calculation of (iv) MRI biomarker maps for microvascular architecture including microvessel density (MVD), the vessel size index (VSI, i.e., microvessel radius) [12] as well as for neovascularization activity represented by the microvessel type indicator (MTI) [9] and (v) macro- and microvascular cerebral blood volume (CBV and μCBV) [16] (red path in Suppl. Fig. 1). For guidance of interpretation of MTI maps, the more negative the MTI value, the stronger the neovascularization activity.

The qBOLD data processing consisted of four steps: (i) corrections for background fields and stimulated echoes of the R2*- and R2-mapping data, respectively; (ii) calculation of R2*- and R2-maps from the multi-echo MR-relaxometry data; and (iii) calculation of absolute flow (CBF) maps from the GE-EPI DSC perfusion MRI data. (iv) Finally, these data were combined with CBV by using the qBOLD approach in order to create the MRI biomarker maps of oxygen extraction fraction (OEF), cerebral metabolic rate of oxygen (CMRO_2_), and tissue oxygen tension (PO_2_) [15] (blue path in Suppl. Fig. 1).

In summary, the procedure resulted in the MRI biomarker maps for macrovascular (CBV) and microvascular perfusion (μCBV), microvascular architecture (MVD and VSI), neovascularization activity (MTI), and oxygen metabolism (OEF, CMRO_2_ and PO_2_), respectively. All eight biomarker maps are summarized at the bottom of Suppl. Fig. 1.

### Quantitative and Statistical Analysis

For quantitative analysis of MR-based pathophysiological brain tumor characteristics, regions of interest (ROIs) were manually defined by an experienced neuroradiologist, neurosurgeon, and medical physicist in consensus based on features seen in both the CE T1-weighted and FLAIR images. ROIs were circles with a diameter ranging between 10 to 20 mm depending on the size of respective area. Four ROIs were located in the necrotic tumor core, the contrast-enhancing tumor bulk, the peritumoral edema, and the contralateral normal appearing brain tissue (cNAB, predominantly white brain matter). To avoid interference by major vessels or tumors regions extending the cortical surface, these areas were excluded from the ROIs [17]. Thereafter, the MRI biomarker maps were coregistered to CE T1-weighted MR images using the rigid registration algorithm of the VINCI software package (version 4.9, Max-Planck-Institute for Neurologic Research, Cologne, Germany) [18] and the R2-map as an anatomical reference. The ROIs were copied to the MRI biomarker maps and mean values calculated.

Software (SPSS 24, IBM, Chicago, IL, USA) was used for statistical evaluation. Differences in the four areas (necrosis, CE tumor, edema, cNAB) between the patient groups diagnosed with glioblastoma and a brain metastasis were determined using the Mann–Whitney *U* test. *P* values less than 0.05 were considered to indicate statistical significance.

## Results

### Patient Characteristics

A total of 33 patients (18 women, 15 men; mean age 62.0 ± 9.7 years; age range 45.2–82.7 years) with newly diagnosed, untreated brain tumors satisfied the inclusion criteria and received a preoperative MRI scans including the sequences specific to our study protocol. Twenty patients (61%; 11 women, 9 men; 62.7 ± 10.7 years; 45.2–82.7 years) were diagnosed with GB WHO grade IV and 13 patients (39%; 7 women, 6 men; 61.0 ± 8.2 years; 47.0–79.1 years) with a BM. In these latter 13 patients, the BM originated in five patients (38%) from lung cancer, in three patients (23%) from breast cancer, in two patients (15%) from a melanoma, and in one patient (8%) each from renal cancer, a fibrosarcoma, and bladder cancer, respectively. All patients were without steroids at the time of the initial MRI scan. The patient characteristics are summarized in Suppl. Table 1 (see ESM).

### Physiological MRI of Patients with GB and BM

Physiological MRI data acquisition and calculation of biomarker maps of perfusion (CBV and μCBV), microvascular architecture (MVD and VSI), neovascularization activity (MTI), and oxygen metabolism (OEF, CMRO_2_, and PO_2_) were successfully performed for all 33 patients.

**Fig. 1.**
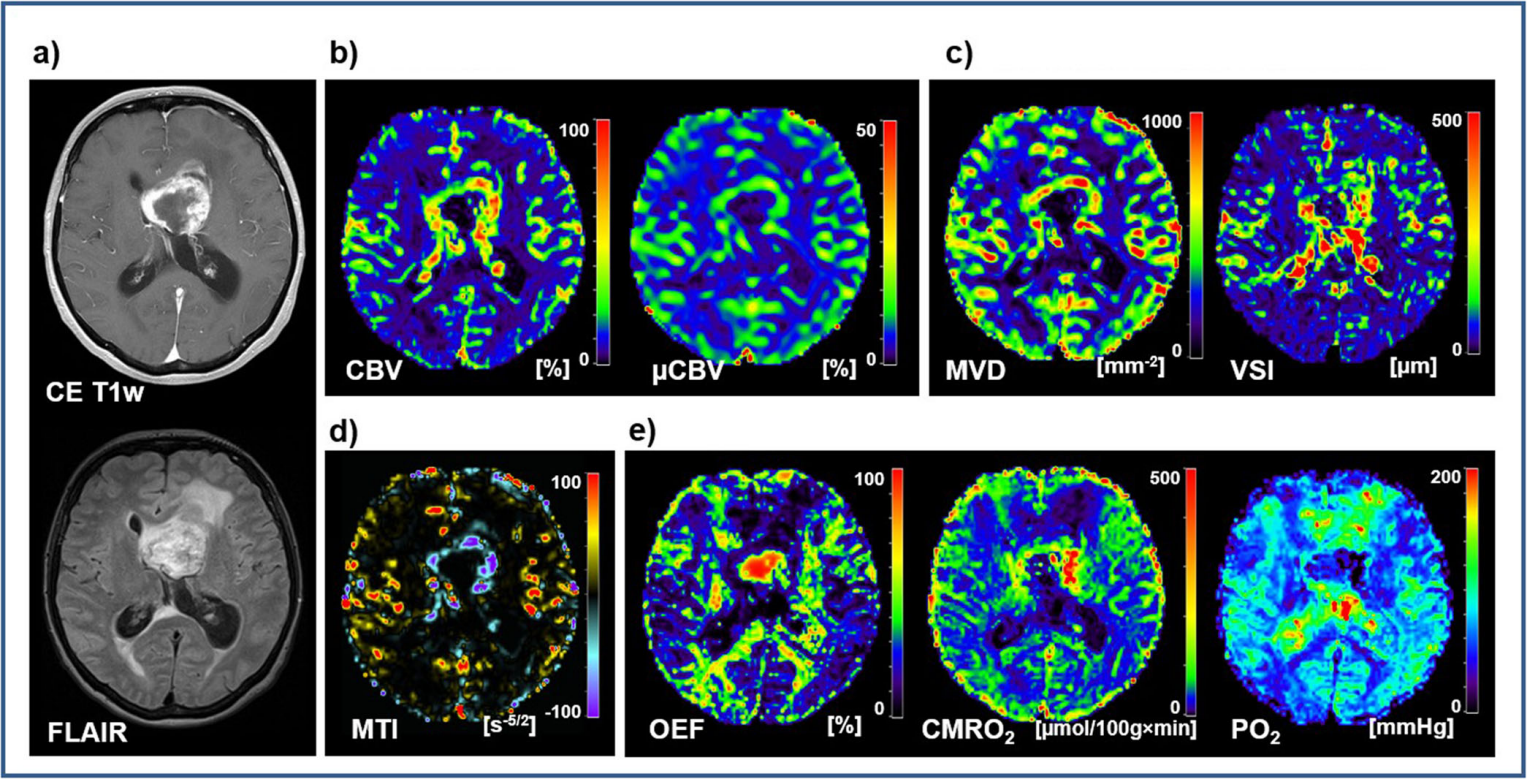
Physiological MR imaging of a patient diagnosed with glioblastoma. **a** Structural MRI data as well as physiological MRI biomarker maps of **b** perfusion including macro- and microvascular cerebral blood volume (CBV and μCBV); **c** microvascular architecture represented by mean vessel (MVD and VSI); **d** neovascularization activity represented by the microvessel type indicator (MTI); and **e** oxygen metabolism including oxygen extraction fraction (OEF), cerebral metabolic rate of oxygen (CMRO_2_), and tissue oxygen tension (PO_2_).

Figure 1 depicts an illustrative case of a patient suffering from GB. The tumor was characterized by extensive central necrosis surrounded by an area of strong contrast enhancement and a moderate perifocal edema. For the necrotic tumor area, as one might expect, physiological MRI data revealed very low perfusion, microvascular density, neovascularization activity, and metabolic rate of oxygen which was associated with a low to very low tissue oxygen tension (PO_2_), i.e., severe hypoxia. The ring of CE viable tumor tissue showed the characteristic hyperperfusion (macro- and microvascular) for this tumor area accompanied by increased microvascular density and vessel size as well as high neovascularization activity. Furthermore, OEF was low (due to hyperperfusion), and CMRO_2_ and PO_2_ were increased respectively. Interestingly, the CE tumor areas with the highest neovascularization activity (high negative MTI values in purple) were found adjacent to the most hypoxic parts (very low PO_2_ values) in region of central necrosis. Peritumoral edema, however, revealed hypoperfusion as well as reduced microvascular architecture and oxygen metabolism (OEF and CMRO_2_) and high levels of oxygen tension (PO_2_).

**Fig. 2.**
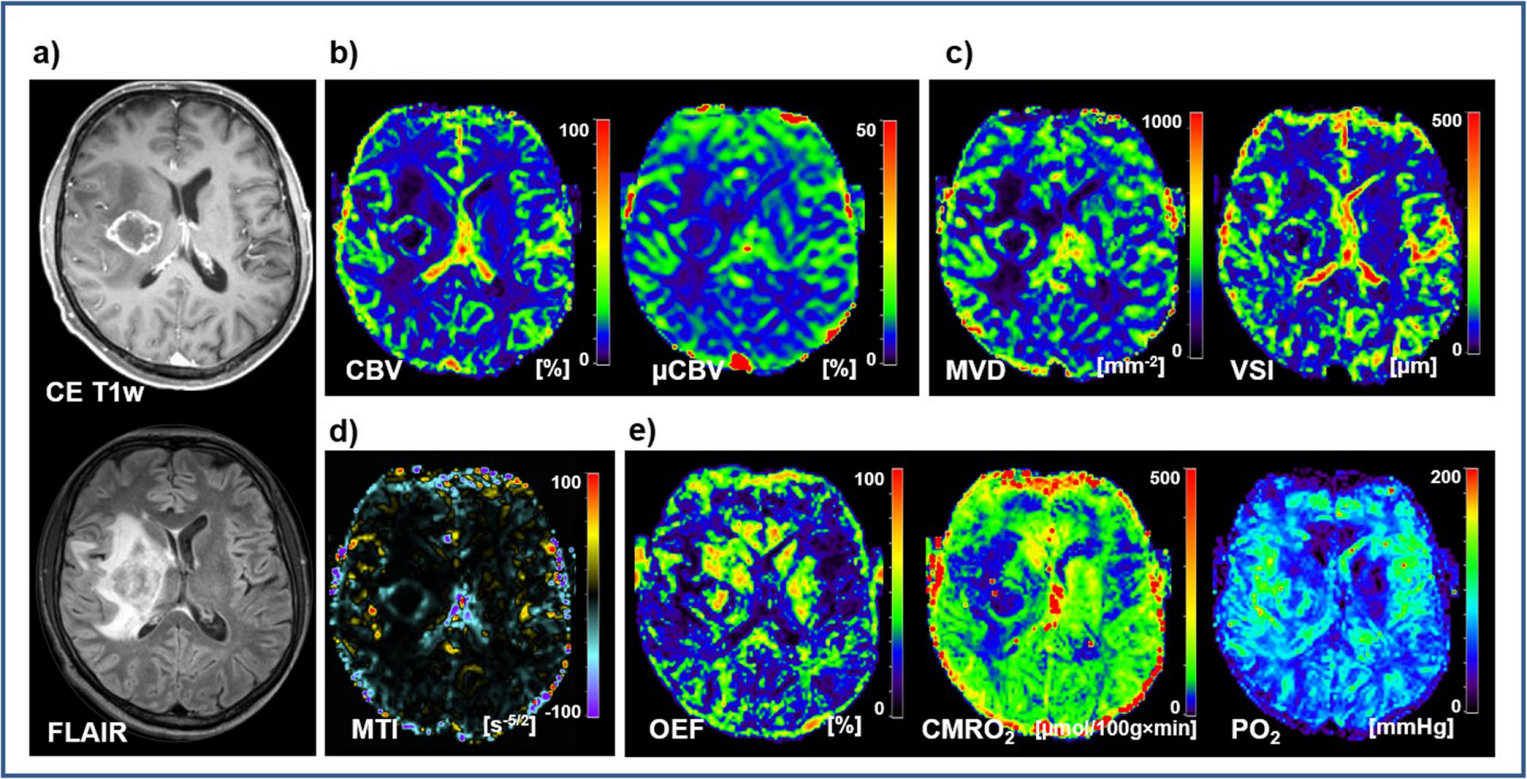
Physiological MR imaging of a patient diagnosed with a brain metastasis originating from bladder cancer. **a** Structural MRI data as well as physiological MRI biomarker maps of **b** perfusion including macro- and microvascular cerebral blood volume (CBV and μCBV); **c** microvascular architecture represented by mean vessel (MVD and VSI); **d** neovascularization activity represented by the microvessel type indicator (MTI); and **e** oxygen metabolism including oxygen extraction fraction (OEF), cerebral metabolic rate of oxygen (CMRO_2_), and tissue oxygen tension (PO_2_).

Figure 2 depicts an illustrative case for a patient suffering from a BM originating from bladder cancer. Structural MRI signal alterations were similar to those of the patient with GB. Physiological MRI data, however, revealed some significant differences in pathophysiology. Tissue oxygen tension (PO_2_) was higher in the area of central necrosis; i.e., the necrotic area was less hypoxic. Neovascularization activity and the metabolic rate of oxygen were lower in the surrounding CE viable tumor ring. The edema around the BM, however, showed higher OEF and lower microvascular perfusion (μCBV) and tissue oxygen tension (PO_2_) compared to that of GB.

### Differences in Physiological MRI Biomarkers Between GB and BM

**Fig. 3.**
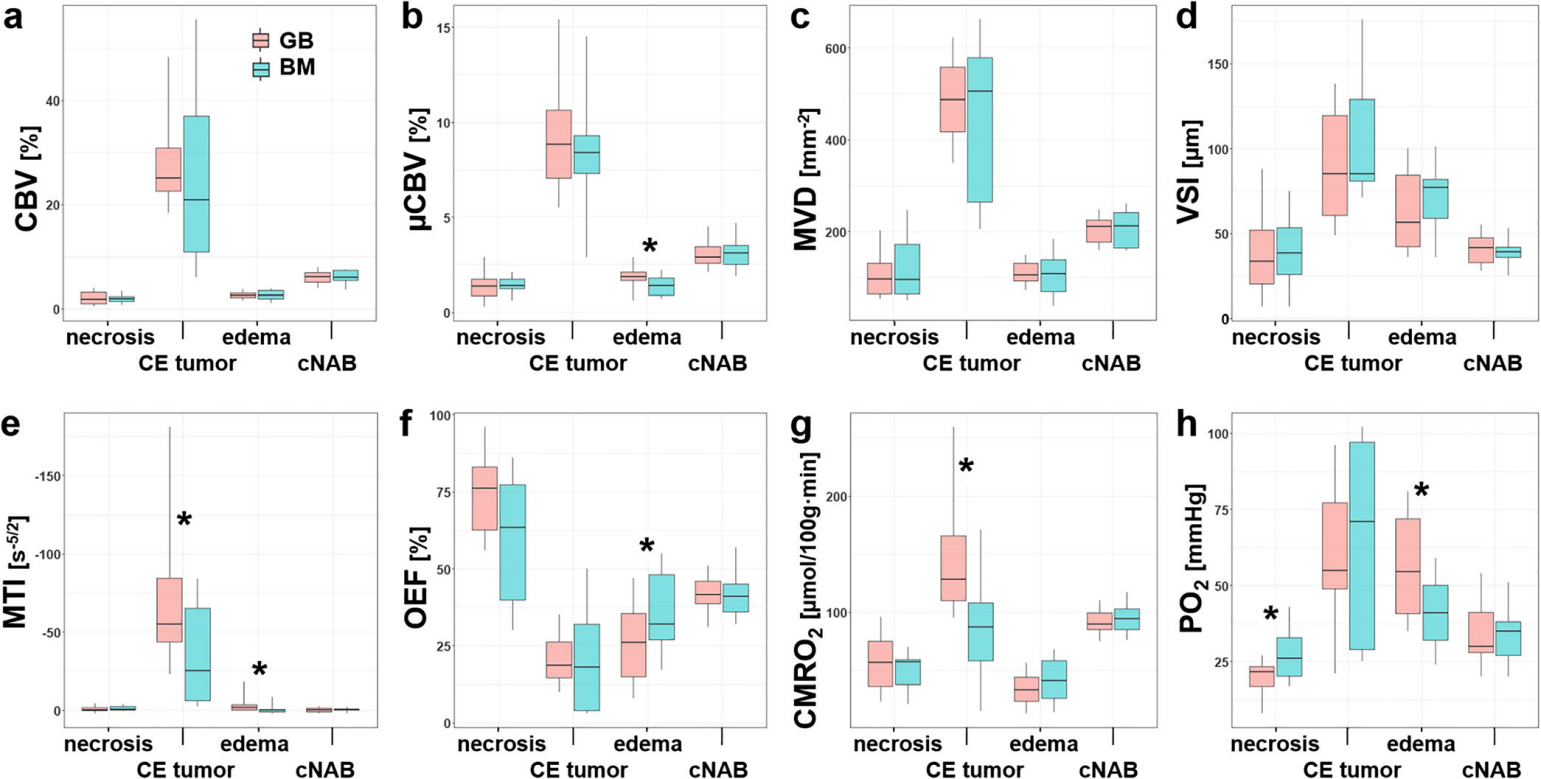
Series of box and whisker plots of MRI biomarker values of **a** macrovascular (conventional) cerebral blood volume (CBV), **b** microvascular cerebral blood volume (μCBV), **c** microvascular density (MVD), **d** vessel size index (VSI, microvessel radius), **e** neovascularization activity represented by microvessel type indicator (MTI), **f** oxygen extraction fraction (OEF), **g** cerebral metabolic rate of oxygen (CMRO_2_), and **h** tissue oxygen tension (PO_2_) for glioblastoma (GB; red boxes) and brain metastasis (BM; cyan boxes), respectively. The asterisks (*) mark significant differences between GB and BM (*P* < 0.05).

The physiological MRI biomarker values in the area of central necrosis, the CE viable tumor, peritumoral edema, and cNAB for all patients in both subgroups (GB and BM) are summarized in Fig. 3 and Table 1, respectively. Here, PO_2_ was the only physiological marker exhibiting significant differences (*P* = 0.036) between GB and BM: the tissue oxygen tension was 30% lower in the necrotic center of GB compared to that of BM. In other words, the area of necrosis in GB was significantly more hypoxic than that of BM. This was associated with a significantly (*P* = 0.043) higher neovascularization activity by 65% and accompanied by a significantly (*P* = 0.004) higher metabolic rate of oxygen by 48% in the CE area of GB. However, the most statistically significant differences in physiological MRI biomarkers between GB and BM were observed in the area of perifocal edema. In the area of perilesional edema of GB, microvascular perfusion (μCBV) was 30% higher (*P* = 0.022), and neovascularization activity was 93% higher (i.e., MTI significantly lower, *P* = 0.018) compared to that of BM. This neovascularization activity in the region of perilesional edema of GB, however, was lower in comparison to that in the CE tumor area. Furthermore, surrounding edematous tissue in patients with GB showed significantly lower OEF (32%; *P* = 0.040) and higher PO_2_ (33%; *P* = 0.020) in comparison to BM.

**Table 1.** Overview of the physiological MRI biomarker values

		Necrosis	CE tumor	Edema	cNAB
CBV	GB	2.0 ± 1.2	28.4 ± 9.4	2.6 ± 0.6	6.0 ± 1.2
[%]	BM	2.0 ± 0.8	25.8 ± 17.4	2.7 ± 0.9	6.0 ± 1.3
μCBV	GB	1.3 ± 0.7	9.3 ± 2.5	1.9 ± 0.5	3.0 ± 0.7
[%]	BM	1.4 ± 0.4	8.4 ± 3.2	1.4 ± 0.5*	3.1 ± 0.8
MVD	GB	106 ± 49	489 ± 81	109 ± 24	204 ± 27
[mm-2]	BM	119 ± 66	439 ± 170	104 ± 47	209 ± 38
VSI	GB	40 ± 27	89 ± 29	62 ± 22	42 ± 9
[μm]	BM	40 ± 20	110 ± 38	73 ± 20	40 ± 8
MTI	GB	−0.9 ± 1.9	−73.2 ± 44.8	−3.3 ± 4.6	−0.2 ± 1.3
[s-5/2]	BM	−1.4 ± 1.6	−37.4 ± 32.2*	−1.2 ± 3.3*	−0.5 ± 1.1
OEF	GB	74 ± 13	21 ± 8	26 ± 12	42 ± 5
[%]	BM	60 ± 21	19 ± 17	36 ± 14*	42 ± 8
CMRO_2_	GB	57 ± 24	148 ± 55	34 ± 13	91 ± 10
μmol/100g×min	BM	50 ± 15	91 ± 45*	44 ± 19	95 ± 13
PO_2_	GB	20 ± 5	59 ± 22	57 ± 17	35 ± 10
[mmHg]	BM	27 ± 9*	66 ± 32	41 ± 12*	34 ± 10

*CBV* macrovascular cerebral blood volume, *μCBV* microvascular cerebral blood volume, *MVD* microvessel density, *VSI* vessel size index, *MTI* microvessel type indicator, *OEF* extraction fraction, *CMRO*_*2*_ cerebral metabolic rate of oxygen, *tissue PO*_*2*_ tissue oxygen tension, *GB* glioblastoma, *BM* brain metastasis

*Values for brain metastasis marked with an asterisk were significant (*P* < 0.05) different to those for glioblastoma

## Discussion

Average life expectancy of patients with melanoma, lung, breast, and other cancers has increased significantly in the past decade as a result of progress in diagnostics and improved therapeutic agents and treatment protocols. Both brain metastases of these malignancies as well GB frequently appear to be rather similar in conventional MRI scans, which represent the current gold standard in diagnostic imaging due to superior soft tissue contrast with correspondingly excellent representation of anatomical details of brain lesions.

Since treatment strategies for each entity differ significantly from one another, initial preoperative discrimination between BM and GB is of great interest in the possible avoidance of potentially unnecessary neurosurgical intervention in patients with BM. Both GB and BM typically present as well-defined, ring-enhancing space occupying lesions with an area of central necrosis in contrast-enhanced T1-weighted sequences accompanied by an inhomogeneous signal intensity associated with peritumoral edema in T2-weighted sequences [8]. A number of previously published studies demonstrated diagnostic uncertainties and a limited ability of conventional MR imaging to distinguish GB from BM [19–21].

In this study, we extended our conventional MR imaging protocol with a physiological MR imaging approach that required 7 min of extra time for data acquisition. The physiological MR imaging biomarker maps revealed several pathophysiological differences between BM and GB.

The central necrosis in GB was more hypoxic compared to BM due the highly proliferative nature of GB characterized by rapid progression. The GB tumor mass expands faster than the formation of new tumor vasculature, resulting in an avascular region with an insufficient oxygen supply and corresponding hypoxia. The structural and functional abnormalities of the newly formed microcirculation and increased diffusion distances between vessels in GB accentuate and propagate the further development of tumoral hypoxia and necrosis. Consequently, as shown in our previous work [14], GB patients with a necrotic/hypoxic dominant phenotype have a significantly shorter progression-free survival in comparison to the glycolytic dominated phenotype. Other authors [22, 23] found a strong correlation between tumor hypoxia and poor prognosis as well as with increased resistance to chemotherapy.

The CE tumor tissue of GB showed a significantly higher neovascularization activity in comparison to BM. The physiological MRI biomarkers for macro- and microcirculation (perfusion and vascular architecture) of GB and BM, however, showed no statistically significant differences in CE region. Remarkably enough, in GB the areas with the highest neovascularization activity (MTI values) as evidenced by CE were found in close vicinity to the most hypoxic parts of the central necrosis. These hotspots may be the reason for the higher neovascularization activity of GB. This could be explained through the activation of the pro-angiogenic factors, such as vascular endothelial growth factor A (VEGF) as a result of the hypoxia-inducible factor-1α (HIF-1α) accumulation in the hypoxic area of GB [24]. Previous studies have demonstrated that CE tumor areas show high tumor cell viability and activity [25, 26]. Gill et al [27] analyzed MRI-guided biopsies from enhancing and non-enhancing parts of GB. The samples from the CE tumor region had significantly higher tumor cell density compared to non-CE regions. This region is characterized with more pronounced destruction of the blood-brain barrier and increased vascularity as a consequence of high neovascularization activity. Furthermore, as shown in several previous studies [28, 29], the overall survival of patients with GB is associated with maximum possible resection of the CE areas of the tumor. In summary, both GB and BM similarly feature high vascularity, abnormal capillary permeability, and breakdown of the blood-brain barrier. Therefore, as further supported by our findings for microvascular perfusion and vascular architecture, biomarkers for perfusion (e.g., CBV) commonly used in clinical routine are not very useful in differentiating between GB and BM [20]. A highly hypoxic central necrosis and adjacent hotspots of high neovascularization activity in the case of GB might be more suitable for this purpose.

Additionally, in the CE area of GB, the metabolic rate of oxygen (CMRO_2_) was significantly elevated in comparison to BM, which might be explained by the high aggressive and proliferative malignancy of GB. Furthermore, the high metabolic turnover rate of GB requires intensive angiogenesis in order to maintain the high metabolic demand. Our findings with regard to GB oxygen metabolism concur that of previously published studies [30, 31].

The most significant and distinct differences in the physiological MRI biomarkers of the two tumor entities were identified in the region of perilesional edema and included μCBV, MTI, OEF, and PO_2_. This was not surprising taking into account the differences in pathophysiological and histopathological mechanisms of edema in GB and BM. GB grows in an invasive manner and is characterized by angiogenesis beyond the CE tumor margins. The peritumoral edema in GB hence consists of a heterogenous mixture of vasogenic, extensive infiltration of glioma cells and neoplastic microvascular hyperplasia and is associated with glial alterations in vital brain tissue. Additionally, some authors reported structural defects in endothelial tight junctions around GB [32, 33]. BM is characterized by expansile growth, and the perilesional edema represents edematous but intrinsically normal brain parenchyma without histological evidence of infiltration by tumor cells. The edema is a purely vasogenic by nature, rich in plasma protein, and a result of uncontrolled leakage of blood plasma into the interstitial compartment from leaky capillaries. This is also supported by the clinical observation that peritumoral T2 signal abnormalities in conventional MRI scans disappear completely following BM resection.

It should be noted that microvascular performance (μCBV) is already a validated and standardized imaging parameter for perfusion MRI techniques with high diagnostic value. Edema secondary to GB is characterized by significantly higher μCBV than that of BM. The decreased microvascular perfusion in the perilesional edema of BM is believed to be due to local compression of the capillaries by the vasogenic edema itself. This is supported by some animal studies showing that cerebral perfusion in edematous tissue was decreased by local compression of the microcirculation by extravasated fluid [34]. On the other hand, increased μCBV in GB is a result of tumor cell infiltration and microvascular hyperplasia. Edematous tissue of BM is more hypoxic in comparison to GB as consequence of the decreased microperfusion.

We found statistically significantly differences in oxygen metabolism in the perilesional edema of the two tumor entities, namely, higher PO_2_ and lower OEF in GB. The neovascularization activity in perilesional edema of GB was also higher too as opposed to that of BM. A possible explanation for these findings is that the perilesional tissue edema surrounding GB is already infiltrated with tumor cells which adapt angioneogenesis to match their increasing metabolic requirement. The MTI reflect the nature of GB as a tumor with a high aggressiveness, malignancy, and pronounced recruitment potential.

We would nevertheless like to point out some limitation of our present study. This was a retrospective study with a relatively small number of patients, the study design of which can introduce unknown bias. The values for the physiological MRI biomarkers showed large overlaps between the subgroups of patients suffering from GB and BM which, additionally, had small sample sizes. This did not allow to define any clinically relevant thresholds nor to assess statistical measures of the diagnostic performance (e.g., specificity or sensitivity). Furthermore, due to the heterogeneity of histologies in the BM patient subgroup (six different primaries), we were not able to provide any comparison between different types of BM. However, this was not the purpose of our study. Future validation of our results would therefore mandate a large prospective study. There was no direct correlation between histological and physiological MTI biomarkers in different tumor areas, possibly because serial biopsy sampling using a neuronavigation system would be required to this end. Another limited factor was the limited variety of metastases studied. The physiological MRI biomarkers for BM were not divided into groups according the histopathological origins due to the small number of patients.

## Conclusions

We performed a systematic analysis of the physiological MRI biomarkers of hypoxia, microvascular architecture, and neovascularization activity in order to differentiate GB from BM in patients with solitary enhancing brain mass in conventional MR scans. Statistically significant differences in physiological MRI biomarkers (μCBV, MTI, OEF, PO_2_) between GB and BM were found in the perilesional edema beyond the CE tumor margins, providing reliable and reproducible results due to its homogeneity and the difference in histology and pathophysiology. Our study therefore demonstrates that physiological MRI biomarkers can successfully distinguish GB and BM from each other.

## Supplementary Information

ESM 1 (DOCX 977 kb)
