# Supplementary material for: Physiological MRI Biomarkers in the Differentiation Between Glioblastomas and Solitary Brain Metastases
Source: Mol Imaging Biol. 2021 Apr 23;23(5):787–95. doi: 10.1007/s11307-021-01604-1 (PMC8410731; doi:10.1007/s11307-021-01604-1)
Supplement: Supplementary file 1 — (DOCX 977 kb) [file 11307_2021_1604_MOESM1_ESM.docx]

**Electronic Supplementary Material**

Physiological MRI biomarkers in the differentiation between glioblastomas and solitary brain metastases

**Journal: Molecular Imaging and Biology**

Elisabeth Heynold^1^, Max Zimmermann^2^, Nirjhar Hore^1^, Michael Buchfelder^1^, Arnd Doerfler^3^, Andreas Stadlbauer^1,4#^ and Natalia Kremenevski^1,#,^*

^1^ Department of Neurosurgery, Friedrich-Alexander University (FAU) Erlangen-Nürnberg, Erlangen, Germany

^2^ Department of Preclinical Imaging and Radiopharmacy, University of Tübingen, Tübingen, Germany

^3^ Department of Neuroradiology, Friedrich-Alexander University (FAU) Erlangen-Nürnberg, Erlangen, Germany

^4^ Institute of Medical Radiology, University Clinic of St. Pölten, Karl Landsteiner University of Health Sciences, St. Pölten, Austria

# shared last authorship

***** Correspondence: Natalia.Kremenevski@uk-erlangen.de; Tel.: +49-9131-8544745

**Supplementary Table 1. Patient characteristics**

| **ID** | **age** | **gender** | **histopathology** | **location** |
| --- | --- | --- | --- | --- |
| 1 | 66.9 | f | GB WHO°IV, IDH wt | left frontal |
| 2 | 79.1 | f | BM from breast Ca | left cerebellar |
| 3 | 46.0 | m | GB WHO°IV, IDH wt | left frontal |
| 4 | 62.1 | f | GB WHO°IV, IDH wt | right parietal |
| 5 | 82.7 | m | GB WHO°IV, IDH wt | left parietal |
| 6 | 63.2 | f | GB WHO°IV, IDH wt | intraventricular/corpus callosum |
| 7 | 47.0 | f | BM from breast Ca | left frontal |
| 8 | 57.9 | m | BM from lung Ca | right frontal |
| 9 | 49.9 | m | GB WHO°IV, IDH wt | right parieto-temporal |
| 10 | 64.7 | f | GB WHO°IV, IDH mut | right parieto-temporal |
| 11 | 61.3 | m | GB WHO°IV, IDH wt | right frontal |
| 12 | 52.5 | m | GB WHO°IV, IDH wt | left frontal |
| 13 | 62.3 | f | BM from breast Ca | right parietal |
| 14 | 69.9 | f | GB WHO°IV, IDH mut | right parietal |
| 15 | 60.2 | m | BM from renal Ca | left cerebellar |
| 16 | 59.1 | m | GB WHO°IV, IDH wt | right parietal |
| 17 | 51.2 | f | BM from lung Ca | right parietal |
| 18 | 77.7 | f | GB WHO°IV, IDH wt | left parietal |
| 19 | 74.1 | m | GB WHO°IV, IDH wt | left frontal |
| 20 | 53.6 | f | GB WHO°IV, IDH wt | left parietal |
| 21 | 55.0 | f | BM from lung Ca | right frontal |
| 22 | 63.1 | m | BM from fibrosarcoma | left fronto-temporal |
| 23 | 75.5 | f | GB WHO°IV, IDH wt | right parieto-occipital |
| 24 | 45.2 | f | GB WHO°IV, IDH wt | left fronto-temporal |
| 25 | 61.2 | f | BM from lung Ca | left frontal |
| 26 | 49.9 | m | GB WHO°IV, IDH wt | right frontal |
| 27 | 57.5 | m | BM from bladder Ca | right frontal |
| 28 | 70.9 | m | GB WHO°IV, IDH wt | right parietal |
| 29 | 64.9 | f | BM from lung Ca | left parietal |
| 30 | 64.1 | f | GB WHO°IV, IDH wt | left frontal |
| 31 | 71.7 | m | BM from melanoma | right parietal |
| 32 | 64.5 | f | GB WHO°IV, IDH wt | right frontal |
| 33 | 62.1 | m | BM from melanoma | right frontal |

m, male; f, female; GB, glioblastoma; IDH, isocitrate dehydrogenase gene; wt, wildtype; mut, mutated; BM, brain metastasis; Ca, carcinoma.

**
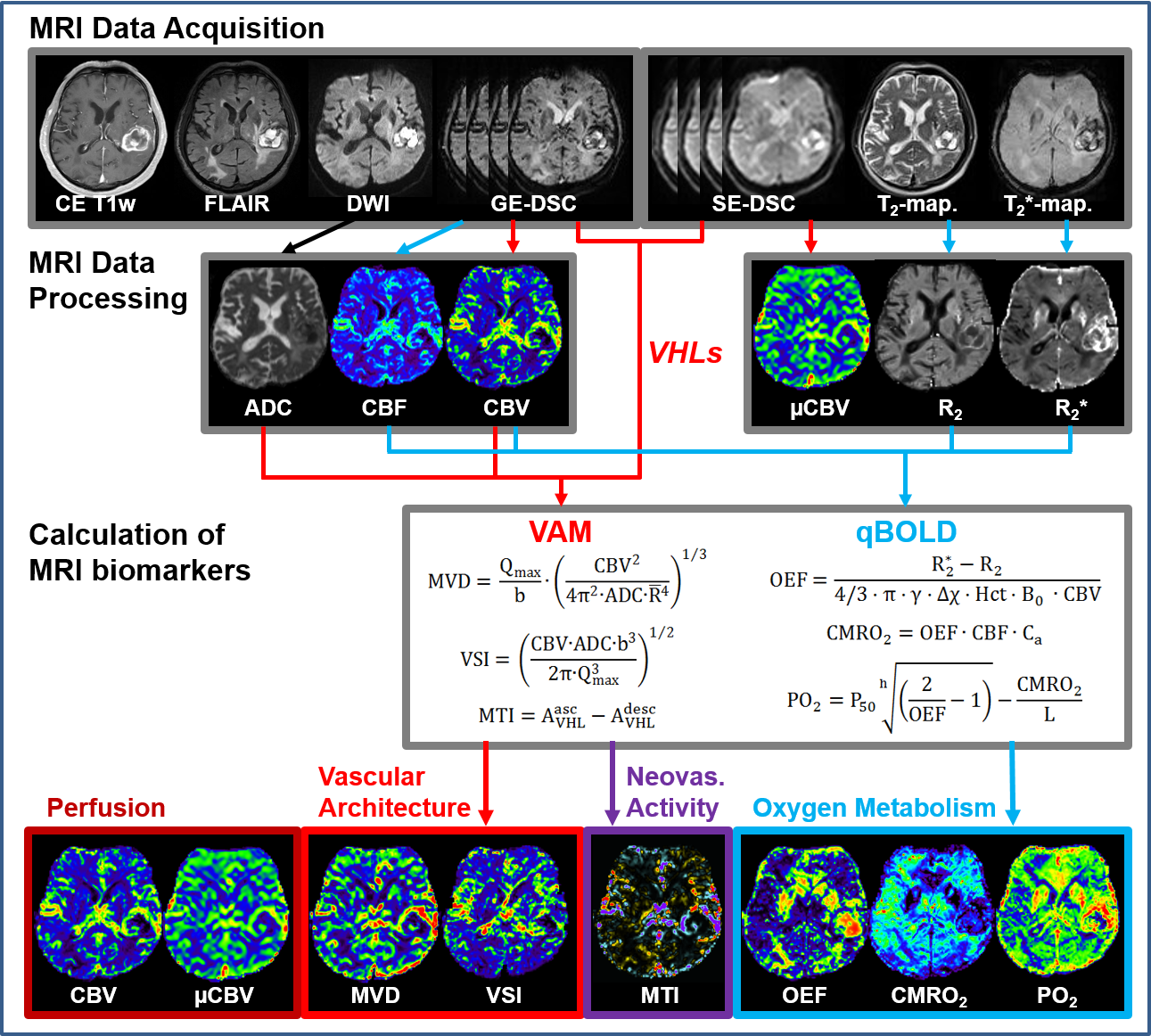
**

**Supplementary Figure 1.** Pipeline for MRI data processing and calculation of MRI biomarker maps described with the data of a patient suffering from a GB. The VAM data processing (red lines) consisted of five steps: (**i**) Correction for remaining contrast agent extravasation was performed as described previously [1–3]; (**ii**) fitting of the first bolus curves for each voxel of the GE- and SE-DSC perfusion MRI data with a previously described gamma-variate function [4]; (**iii**) Calculation of the ∆R_2,GE_ versus (∆R_2,SE_)^3/2^ diagram [5], the so-called vascular hysteresis loop (VHL) [3, 6]. These data were subsequently used for (**iv**) calculation of maps for microvascular architecture including microvessel density (MVD), the vessel size index (VSI, i.e. microvessel radius) [7] as well as for neovascularization activity represented by the microvessel type indicator (MTI) [3]. For MVD and VSI, we used the following equations:

$$\text{MVD = }\frac{\text{Q}_{\text{max}}}{\text{b}}\text{∙}\left( \frac{\text{CBV}^{\text{2}}}{\text{4}\text{π}^{\text{2}}\text{ }\text{∙}\text{ }\text{ADC}\text{ }\text{∙}\text{ }{\bar{\text{R}}}^{\text{4}}} \right)^{\text{1}/\text{3}}$$

and

$$\text{VSI = }\left( \frac{\text{CBV}\text{ }\text{∙}\text{ }\text{ADC}\text{ }\text{∙}\text{ }\text{b}^{\text{3}}}{\text{2π}\text{ }\text{∙}\text{ }\text{Q}_{\text{max}}^{\text{3}}} \right)^{\text{1}/\text{2}}$$

with Q_max_ = max[∆R_2,GE_]/max[(∆R_2,GE_)^3/2^]; ADC = apparent diffusion coefficient which was calculated from the DWI data; $\bar{\text{R}}$ ≈ 3.0 μm is the mean vessel lumen radius and b is a numerical constant (b = 1.6781) [7]. MTI was defined as the area of the VHL signed with the rotational direction of the VHL, i.e. a clockwise VHL-direction was identified with a plus-sign, and a counter-clockwise VHL-direction was identified with a minus-sign [3]. In a final step (**v**) the map for the microvascular cerebral blood volume (μCBV) was calculated from the SE-DSC perfusion MRI data via a separate automatic identification of AIFs [8]. In summary, this resulted in the MRI biomarker maps of perfusion (CBV and µCBV), microvascular architecture (MVD and VSI), and neovascularization activity (MTI), respectively.

The qBOLD data processing (blue lines) consisted of four steps: (**i**) Corrections for background fields of the R_2_*-mapping data [9] and for stimulated echoes of the R_2_-mapping data [10]. (**ii**) Calculation of R_2_*- and R_2_-maps from the multi-echo relaxometry data, and (**iii**) of absolute cerebral blood volume (CBV) and flow (CBF) maps from the GE-DSC perfusion MRI data via automatic identification of arterial input functions (AIFs) [8, 11]. In the final step (**iv**) MRI biomarker maps of oxygen metabolism including oxygen extraction fraction (OEF), cerebral metabolic rate of oxygen (CMRO_2_) [12], and the tissue oxygen tension (PO_2_) [13, 14] were calculated using the following equations:

$$\text{OEF}\text{ }\text{=}\text{ }\frac{\text{R}_{\text{2}\text{ }}^{\text{*}}-\text{ }\text{R}_{\text{2}}}{\frac{\text{4}}{\text{3}}\text{ }\text{·}\text{ }\text{π}\text{ }\text{·}\text{ }\text{γ}\text{ }\text{·}\text{ }\text{Δχ}\text{ }\text{·}\text{ }\text{Hct}\text{ }\text{·}\text{ }\text{B}_{\text{0}}\text{ ∙}\text{ }\text{CBV}}$$

with γ (2.67502·10^8^ rad/s/T) is the nuclear gyromagnetic ratio; Δχ = 0.264·10^-6^ is the difference between the magnetic susceptibilities of fully oxygenated and fully deoxygenated haemoglobin; Hct = 0.42·0.85 is the microvascular hematocrit fraction, whereby the factor 0.85 stands for a correction factor of systemic Hct for small vessels;

$$\text{CMRO}_{\text{2}}\text{ }\text{=}\text{ }\text{OEF}\text{ }\text{∙}\text{ }\text{CBF}\text{ }\text{∙}\text{ }\text{C}_{\text{a}}$$

where C_a_ = 8.68 mmol/ml is the arterial blood oxygen content [15]; and

$$\text{PO}_{\text{2}}\text{ }\text{=}\text{ }\text{P}_{\text{50}}\cdot\sqrt[\text{h}]{\left( \frac{\text{2}}{\text{OEF}} -\text{ }\text{1} \right)} -\text{ }\frac{\text{CMRO}_{\text{2}}}{\text{L}}$$

where P_50_ is the hemoglobin half-saturation tension of oxygen (27 mmHg), h is the Hill coefficient of oxygen binding to hemoglobin (2.7), and L (4.4 µmol/mmHg per minute) is the tissue oxygen conductivity as defined by Vafaee and Gjedde [16] OEF describes the percent of the oxygen removed from the blood by tissue, CMRO_2_ is the rate of oxygen consumed by the tissue in µmol/100g×minute, and PO_2_ (in mmHg) reflects the balance between the delivery and consumption of oxygen.

**References for Supplementary Figure 1**

1. Boxerman JL, Prah DE, Paulson ES, et al (2012) The role of preload and leakage correction in gadolinium-based cerebral blood volume estimation determined by comparison with MION as a criterion standard. Am J Neuroradiol 33:1081–1087

2. Boxerman JL, Schmainda KM, Weisskoff RM (2006) Relative cerebral blood volume maps corrected for contrast agent extravasation significantly correlate with glioma tumor grade, whereas uncorrected maps do not. Am J Neuroradiol 27:859–867

3. Stadlbauer A, Zimmermann M, Heinz G, et al (2017) Magnetic resonance imaging biomarkers for clinical routine assessment of microvascular architecture in glioma. J Cereb Blood Flow Metab 37:632–643

4. Ducreux D, Buvat I, Meder JF, et al (2006) Perfusion-weighted MR imaging studies in brain hypervascular diseases: comparison of arterial input function extractions for perfusion measurement. AJNR Am J Neuroradiol 27:1059–1069

5. Xu C, Kiselev VG, Möller HE, Fiebach JB (2013) Dynamic hysteresis between gradient echo and spin echo attenuations in dynamic susceptibility contrast imaging. Magn Reson Med 69:981–991

6. Stadlbauer A, Zimmermann M, Oberndorfer S, et al (2017) Vascular Hysteresis Loops and Vascular Architecture Mapping in Patients with Glioblastoma treated with Antiangiogenic Therapy. Sci Rep 7:1–12

7. Jensen JH, Lu H, Inglese M (2006) Microvessel density estimation in the human brain by means of dynamic contrast-enhanced echo-planar imaging. Magn Reson Med 56:1145–1150

8. Bjornerud A, Emblem KE (2010) A fully automated method for quantitative cerebral hemodynamic analysis using DSC-MRI. J Cereb Blood Flow Metab 30:1066–1078

9. Preibisch C, Volz S, Anti S, Deichmann R (2008) Exponential excitation pulses for improved water content mapping in the presence of background gradients. Magn Reson Med 60:908–916

10. Prasloski T, Mädler B, Xiang QS, et al (2012) Applications of stimulated echo correction to multicomponent T2 analysis. Magn Reson Med 67:1803–1814

11. Smith AM, Grandin CB, Duprez T, et al (2000) Whole Brain Quantitative CBF, CBV, and MTT Measurements Using MRI Bolus Tracking: Implementation and Application to Data Acquired From Hyperacute Stroke Patients. J Magn Reson Imaging 12:400–410

12. Christen T, Schmiedeskamp H, Straka M, et al (2012) Measuring brain oxygenation in humans using a multiparametric quantitative blood oxygenation level dependent MRI approach. Magn Reson Med 68:905–911

13. Vafaee MS, Vang K, Bergersen LH, Gjedde A (2012) Oxygen consumption and blood flow coupling in human motor cortex during intense finger tapping: implication for a role of lactate. J Cereb Blood Flow Metab 32:1859–68

14. Gjedde A (2002) Cerebral blood flow change in arterial hypoxemia is consistent with negligible oxygen tension in brain mitochondria. Neuroimage 17:1876–1881

15. Kennan RP, Zhong J, Gore JC (1994) Intravascular susceptibility contrast mechanisms in tissues. Magn Reson Med 31:9–21

16. Vafaee MS, Gjedde A (2000) Model of blood-brain transfer of oxygen explains nonlinear flow-metabolism coupling during stimulation of visual cortex. J Cereb blood flow Metab 20:747–754
